# Burnout Among Private Security Staff in Serbia: A Multicentic Cross-Sectional Study

**DOI:** 10.3389/fpubh.2021.622163

**Published:** 2021-05-07

**Authors:** Dejan R. Veljković, Natasa K. Rancic, Momčilo R. Mirković, Ljiljana M. Kulić, Veroslava V. Stanković, Ljubomir S. Stefanović, Marko M. Stojanović, Miloš V. Mirković, Slađana M. Ðurić, Danijela Z. Ilić

**Affiliations:** ^1^Other, Kraljevo, Serbia; ^2^University of Niš, Niš, Serbia; ^3^Faculty of Medicine, University of Prishtina, Prishtina, Serbia; ^4^Other, Belgrade, Serbia; ^5^Institute of Public Health Nis, Nis, Serbia

**Keywords:** burnout, predictors, prevntion, working staff, MBI questionnaire

## Abstract

**Background:** Burnout is a special a state of physical or emotional exhaustion that also involves a sense of reduced accomplishment and loss of personal identity.

**Objectives:** To evaluate the predictors of burnout among work staff in the seven private agencies for support and defense of persons and their property.

**Material and Method:** We performed a multicentric cross-sectional study that involved representative sample of working staff from Agencies of Private Security in Central Serbia. Burnout was assessed using Maslach Burnout Inventory- (MBI)-Human Services Survey.

**Results:** A total number of participants were 353 (330 men and 23 women). Measured level of burnout as assessed by high emotional exhaustion, high depersonalization, and low personal accomplishment was 66.3, 82.4, and 13.4%, respectively. We identified that female gender, younger age, shorter work experience, working in shifts, working 12 h a day and more than 8–12 h a day as well as dissatisfaction with working conditions. Work in shifts, working 12 h a day and more than 8–12 h a day and dissatisfied with dissatisfaction with working conditions significantly increase the risk of total burnout.

**Conclusion:** Our results showed that significant predictors for the development of burnout syndrome were female gender, younger age, shorter work experience, working in shifts, as well as dissatisfaction with working conditions.

## Introduction

Burnout is a specific syndrome that is a consequence of prolonged exposal to occupational stress, and it is primarily specific for occupations featured by working with people in emotionally challenging situations (1). There is no standard definition of this syndrome, and the most often quoted is the one proposed by Maslach and Jakson (2) that defines burnout as “a psychological syndrome involving emotional exhaustion, depersonalization, and a diminished sense of personal accomplishment that occurred among various professionals who work with other people in challenging situations.”

There is a lot of research on the burnout syndrome of police officers around the world (3–10), and the occupation of a police officer is undoubtedly one of the most stressful ones (11). In addition to securing public order and peace and traffic regulation, similar work is also performed by employees who secure other people and their property.

For professional staff who provides security of individuals and their property and who needs to spend a lot of time in intensive interaction with the clients and focus on current issues, chronic stress can be emotionally draining and it poses a risk of “burnout.” Burnout usually takes place about a year after a person starts working in an institution (12, 13). Three key aspects of the burnout syndrome are emotional exhaustion, depersonalization, and a diminished sense of personal accomplishment, which occur as a response to chronic stress at jobs related to direct working with people (14–17).

Emotional exhaustion relates to an individual's assessment that their emotional and physical strength is exhausted over the limits, and the symptoms often manifest as fatigue, headache, insomnia, and appetite disorder. The emotional exhaustion symptoms are central and they are accompanied by depersonalisation and a diminished sense of personal accomplishment (15, 16). Emotional exhaustion is featured by a sense of emotional overextension as a result of work; depersonalisation is featured by emotional indifference and dehumanization of service recipients, and the diminished sense of personal accomplishment is featured by the sense of professional stagnation, incapability, incompetence, and unfulfillment (17, 18).

Depersonalisation refers to the development of insensitive and cynical attitude toward people who are service recipients, negative attitude toward work, as well as the loss of sense of own identity. The diminished sense of personal accomplishment refers to negative self-assessment of competences and accomplishments at a workplace, and the symptoms are visible as a lack of motivation to work, decline in self-esteem and general productivity (14–17).

Due to professional stress and the arising burnout, the employees are at risk of clinical manifestations of depression, professional impairment, leaving work, alcohol and drugs abuse, suicide, and increased aggression toward others (7, 8). Burnout has serious professional and personal consequences, including lack of professionalism (often sick-leaves, reduced efficiency at work, lack of interest, non-collegiality) (19), and problems in communication with close persons, divorces, losing friends, alienation, aggressiveness privately (20, 21).

It is known that burnout occurs as a result of a complex interplay between individual-demographic risk factors and organizational factors (4). Several individual level factors, including external locus of control, poor self-esteem, and maladaptive coping styles, have been associated with burnout (17, 18).

Various studies have dealt with the research of external factors, and particularly with the so-called burnout external triggers such as the organizational structure and the effect of the social environment factors (14, 15, 19–22) dealing with the key issue—why in the same working conditions one employee experiences the burnout syndrome and the other does not.

The objective of the paper was to identify and analyse predictors for burnout among the staff who secures individuals and their property in Central Serbia.

## Materials and Methods

### Study Design

The study was conducted in the population of Central Serbia, excluding the territory of Autonomous Province Vojvodina and Autonomous Province of Kosovo and Metohija. A multicentric cross-sectional study was applied that included staff employed at private security agencies in seven cities in Central Serbia. The study was conducted on a representative sample whose size was determined by https://www.statisticshowto.com/probability-and-statistics/find-sample-size/.

The representative sample size was 439, the study strength is 80% and type 1 error probability α of 0.05. The study included 353 questionnaires that were completely filled out.

The study was performed in the period from March 3, 2019 to April 30, 2019.

#### Study Inclusion Criteria

Adults 18–65 years of age, citizens of the Republic of Serbia, full-time employees, licensed to work in private security and working longer than 12 months.

#### Exclusion Criteria

Staff in the process of obtaining the license, discontinuity at work longer than 1 year, long-term sick leaves or multiple changes of workplace in the past 5 years, respondents recently exposed to major psychophysical trauma regardless of the professional environment (illness or death of a close person, divorce, etc.), refusal to participate in the research.

### Questionnaires

An epidemiological questionnaire has been prepared to gather respondents' descriptive information and a specific questionnaire for burnout syndrome risk level assessment for representatives of service industries (Maslach Burnout Inventory—MBI-HSS), (18).

A semi-structural epidemiological questionnaire with 20 questions has been prepared, specially designed for this research, to gather the basic socio-demographic information (sex, age, marital status and number of children, level of education, length of service, work experience and length of service at any of the managerial positions, shift work, field work, specific features of a workplace).

One question on a three-point Likert scale examined the level of the respondents' satisfaction with the conditions of their work. The respondents were offered a possibility to present in an open question any complaints on the work conditions. Household financial status was assessed based on the housing status, amount of personal income and additional revenues.

The MBI-HSS questionnaire is an internationally accepted burnout measuring standard that measures 3 burnout dimensions and it is often used as a model for the evaluation of validity of other burnout risk assessment scales.

MBI today is considered the most famous and most reliable measuring instrument for burnout syndrome measuring, which is supported by the fact that over 90% of implemented surveys have used this instrument (21). Maslach et al. have originally defined burnout as a psychological syndrome of emotional exhaustion, depersonalisation (later replaced with cynicism construct) and reduced effectiveness or personal accomplishment, which make this scale a multidimensional construct. We have used the questionnaire for staff employed at institutions who are in direct contact with people (Human Services Survey, MBI-HSS) with 22 variables.

MBI-HSS consists of the total of 22 questions that are afterwards used in the calculation of three subscales measuring different occupational burnout aspects:

1. Emotional exhaustion—measures the sense of emotional strain and exhaustion caused by work.
2. Depersonalisation—measures the inexistence of sense and impersonal reaction toward the recipient of services, help, treatment or tutoring, or a sense of discomfort caused by exertion.
3. Personal fulfillment or lack of personal accomplishment—measures the experience of competence and success in working with people, or a sense of competition and job satisfaction.

The Emotional Exhaustion (EE) subscale is made of 9 questions, the Depersonalisation (DP) scale is made of 5 questions and the Personal Accomplishment (PA) scale is made of 8 questions. Each question is made up of a series of statements expressing the degree of agreement with the expressed statements, and the response categories are provided through the 7-point Likert scale from 0 (never) to 6 (every day), or (0—never, 1—once a year or less, 2—once a month and less, 3—a few times a month, 4—once a week, 5—a few times a week, 6—every day).

The total score of each respondent was obtained by summing with a specific key the matrix for each of the three previously mentioned subscales, and the total degree of occupational burnout is represented by a comprehensive scale calculated based on a precise formula (18). The limit values of the calculated subscales are given in Table 1.

**Table 1:** The limit values of the calculated subscales.

**Emotional exhaustion**		**Depersonalisation**		**Personal accomplishment**	
**Level**	**Value**	**Level**	**Value**	**Level**	**Value**
Low	0–16 points	Low	0–6 points	Low	0–31 points
Medium	17–26 points	Medium	7–12 points	Medium	32–38 points
High	27 points and more	High	13 points and more	High	39 points and more

High level of burnout at work is reflected in high scores on the emotional exhaustion and depersonalisation subscales and low scores on the personal accomplishment subscale. This means that high scores on the EE and DP scales contribute to the burnout syndrome, while high scores on the professional accomplishment scale diminishes it (19).

Medium level of occupational burnout syndrome is a reflection of means on all scales.

Low level of burnout at work is reflected in low scores on the emotional exhaustion and depersonalisation subscales and high scores on the personal accomplishment subscale. We cannot make a conclusion about the presence of occupational burnout syndrome if only the PA subscale is observed. The PA scale is relevant only if confirmed with the EE or DP scale.

Licenses for the MBI-HSS questionnaire and the evaluation key, as well as the usage permission were obtained directly from the current license owners—from the SINAPSA EDITION Company according to license no. 2/2018 dated May 9, 2018.

The research was approved by the Ethics Committee of the Faculty of Medicine of the University of Pristina with temporary seat in Kosovska Mitrovica by the Decision No 09-972-1 dated September 10, 2018.

The managers of private security agencies provided written approvals for the research. All the respondents were informed in detail about the research and they signed the consents to participate in the study.

### Statistical Analyses

All the analyses were done in the SPSS-ver. 22 program package. Data were presented as the mean and standard deviation (SD) or as frequencies and proportions. Linear univariate regression analysis was used to find association between assumed predictors. Statistically significant parameters were then included in the multivariate analysis. A multivariate regression analysis was applied to determine the most important predictors of burnout syndrome. The results are presented as Beta coefficients and its *p*-value. The *p*-value below 0.05 (*p* < 0.05) was considered statistically significant.

## Results

A total of 353 respondents (330–93.5% male and 23–6.5% female) participated in the research. The response rate was 80%. The average age of all the respondents was 43.62 ± 11.37. The average age of men was significantly higher than the age of women, 44.09 ± 11.44 vs. 36.91 ± 7.92 (*F* = 8.752; *p* = 0.003).

Basic socio-demographic characteristics and the length of service in regard to the gender structure of the respondents are presented in Table 2.

**Table 2:** Basic socio-demographic characteristics and the length of service in regard to the sex distribution of the respondents.

**Sociodemographic characteristics**		**Number percentage**
**Sex**	Male	330 (93.5%)
	Female	23 (6.5%)
**Marital status**	Married	227 (64.3%)
	Unmarried	87 (24.6%)
	Extramarital union	12 (3.5%)
	Divorced	23 (6.5%)
	Widower/widow	4 (1.1%)
**Number of children**	None	119 (33.7%)
	One	84 (23.8%)
	Two	129 (36.5%)
	Three	20 (5.7%)
	More	1 (0.3%)
**Education**	Three years of high school	103 (29.2%)
	Four years of high school	193 (54.7%)
	High school–two and a half year studies	16 (4.5%)
	High school–three-year vocational or academic studies	14 (4.0%)
	Faculty–basics or master studies	27 (7.6%)
**Workplace**	Administrative worker	6 (1.7%)
	Director	8 (2.3%)
	Coordinator	3 (0.8%)
	Manager	5 (1.4%)
	Control center operator	20 (5.7%)
	Security officer	273 (77.3%)
	Boss of security service	38 (10.8%)
**Work in shifts**	Yes	288 (81.6%)
	No	65 (18.4%)
**Work hours**	Up to 8 h	36 (10.2%)
	8–12 h	277 (78.5%)
	More than 12 h	40 (11.3%)
**Work conditions**	Satisfied	225 (63.7%)
	Not satisfied nor dissatisfied	109 (30.9%)
	Dissatisfied	19 (5.4%)
**Possession of weapons**	Yes	115 (32.6%)
	No	238 (67.4%)
**Housing issue**	Owner of residential building in which I live	239 (67.7%)
	Owner of residential building for which I am paying rent for	15 (4.3%)
	Tenant	99 (28%)
**Monthly income**	Less than minimal wage	9 (2.5%)
	Minimal wage	252 (71.4%)
	Above minimal wage	92 (26.1%)
**Additional income**	Yes	109 (30.9%)
	No	244 (69.1%)

Men represent 93.5% of all the respondents; they were significantly older than women. More than 64.3% of the respondents were married; about one third did not have children 33.7%, and more than a half of the respondents had completed secondary school 54.7%. The least of the employed had a University degree 7.6%.

239 (67.7%) had a settled housing issue. More than 70% of the respondents were in the service and about 10% held managing positions. 81.6% of respondents worked in shifts, and most of the respondents—as much as 277 (78.5%) worked from 8 to 12 h.

More than 60% responded that they are satisfied with the working conditions: Approximately one third (32.6%) is armed at work. The biggest number of the respondents (71.4%) had monthly revenue at the level of a minimum average salary, and 30.9% of the respondents had additional income.

The average length of service of men was 18.43 ± 11.93 and it was significantly higher than the length of service of women 10.08 ± 7.27 years (*F* = 10.969; *p* = 0.001), are presented in Table 3.

**Table 3:** Influence of sex and length of service on the risk of occurance of burnout syndrome in the observed population.

**Variable**		***N***	X^*^	**SD**	**SEM**	**95% C**		**Min**	**Max**
						**Lower**	**Upper**		
Sex	Male	330	44.09	11.44	0.62	42.85	45.33	20.00	69.00
	Female	23	36.91	7.92	1.65	33.48	40.34	20.00	51.00
	***F*** **=** **8.752;** ***p*** **=** **0.003**								
Length of service	Male	330	18.43	11.93	0.65	17.14	19.73	1.00	49.00.0
	Female	23	10.08	7.27	1.51	6.94	13.23	1.00	23.00.0
	***F*** **=** **10.969;** ***p*** **=** **0.001**								

*N, Number of responders*.

X^*^-average value.

Results of multivariate regression analysis in regard to the dependant EE variable are in Table 4.

**Table 4:** Factors affecting the EE variable: results of multiple regression analysis.

**Characteristics**		**B**	**S.E**.	**Wald**	**df**	**Sig**.	**Exp (B)**
Sex	Female	Reference value					
	Male	−1.285	0.630	4.16	1	0.041	0.277
Age		−0.039	0.010	13.70	1	0.000	0.962
Marital status	Married	Reference value					
	Unmarried	1.746	1.163	2.25	1	0.133	5.731
	Extramarital union	1.951	1.178	2.74	1	0.098	7.038
	Divorced	1.435	1.295	1.22	1	0.268	4.200
	Widower/widow	1.925	1.240	2.40	1	0.121	6.857
Number of children	None	Reference value					
	One	0.084	0.306	0.07	1	0.784	1.088
	Two	−0.128	0.268	0.22	1	0.632	0.880
	Three	−0.313	0.496	0.39	1	0.528	0.731
Education	Three years of high school	Reference value					
	Four years of high school	−0.057	0.255	0.05	1	0.822	0.944
	High school—two and a half year studies	0.477	0.613	0.60	1	0.436	1.612
	High school−3-year vocational or academic studies	0.295	0.627	0.22	1	0.638	1.343
	Faculty—basics or master studies	0.860	0.537	2.56	1	0.109	2.364
Length of service		−0.037	0.010	14.25	1	0.000	0.964
Length of service in company		−0.056	0.025	5.13	1	0.024	0.946
Work in shifts	No	Reference value					
	Yes	0.253	0.299	0.71	1	0.398	1.288
Work hours	Up to 8 h	Reference value					
	8–12 h	−0.432	0.405	1.13	1	0.286	0.649
	More than 12 h	−0.693	0.502	1.90	1	0.168	0.500
Management function	No	Reference value					
	Yes	−0.207	0.309	0.45	1	0.502	0.813
Work conditions	Satisfied	Reference value					
	Neither satisfied nor dissatisfied	−0.705	0.243	8.38	1	0.004	0.494
	Dissarisfied	−0.626	0.488	1.64	1	0.199	0.535

Table 5 shows the results of DR scale modeling with the application of multivariate logistic regression analysis in regard to the independent variables as follows: gender, age, marital status, number of children, education, length of service, service in the company, shift work, working hours, managerial function, and working conditions.

**Table 5:** Factors affecting the DP variable: results of multiple regression analysis.

**Characteristics**		**B**	**S.E**.	**Wald**	**df**	**Sig**.	**Exp (B)**
Sex	Female	Reference value					
	Male	−0.373	0.636	0.344	1	0.558	0.689
Age		−0.011	0.012	0.832	1	0.362	0.989
Martial status	Married	Reference value					
	Unmarried	−0.083	0.328	0.064	1	0.801	0.921
	Extramarital union	0.037	0.794	0.002	1	0.963	1.037
	Divorced	−0.292	0.535	0.297	1	0.585	0.747
	Widower/widow						
Number of children	None	Reference value					
	One	0.461	0.414	1.241	1	0.265	1.586
	Two	−0.115	0.328	0.123	1	0.726	0.891
	Three	−0.693	0.544	1.624	1	0.203	0.500
Education	Three years of high school	Reference value					
	Four years of high school	−0.411	0.341	1.452	1	0.228	0.663
	High school—two and a half year studies	−0.383	0.702	0.298	1	0.585	0.682
	High school−3-year vocational or academic studies	−0.550	0.712	0.597	1	0.440	0.577
	Faculty—basics or master studies	−0.368	0.573	0.413	1	0.521	0.692
Length of service		−0.003	0.012	0.071	1	0.790	0.997
Length of service in company		−0.052	0.029	3.31	1	0.069	0.949
Work in shifts	No	Reference value					
	Yes	0.671	0.427	2.46	1	0.116	1.956
Work hours	Up to 8 h	Reference value					
	8–12 h	−0.336	0.506	0.440	1	0.507	0.715
	More than 12 h	−0.090	0.654	0.019	1	0.891	0.914
Management function	No	Reference value					
	Yes	0.048	0.396	0.015	1	0.904	1.049
Work conditions	Satisfied	Reference value					
	Not satisfied nor dissatisfied	−0.219	0.298	0.540	1	0.463	0.804
	Dissatisfied	0.547	0.768	0.506	1	0.477	1.727

The dependent variable “depersonalisation” was transformed into a binary type. The reference category is represented by low and moderate level of depersonalisation. The effect of dependent variables on the independent variable-level depersonalisation has not been determined.

The dependent variable “personal accomplishment” was transformed into a binary type. The reference category is represented by high and moderate level of PA. The gender of the respondents stood out as a significant variable in the dependent PA variable modeling. The cross-ratio of “male” in comparison to the reference category “female” amounts to 2,644 and the significance is at the level of 0.10, while its corresponding 90% confidence interval for the cross-ratio is 0.879−7.954. If a respondent is male, the probability that he will manifest the burnout syndrome in the form of lowered PA is reduced by 164% in comparison to female respondents.

The respondents' education also stood out as a significant variable in the dependent PA variable. The cross-ration of “college” education in comparison to the reference category of “secondary three-year school” amounts to 3.698 and the significance is at the level of 0.10, while its corresponding 90% confidence interval for the cross-ration amounts to 1.237–11.051. If a respondent has college education, the probability that they will manifest the burnout syndrome in the form of lowered PA is reduced by 269% in comparison to the respondents with 3-year secondary school.

Table 6 shows the results of PA subscale modeling with the application of multivariate logistic regression analysis in regard to the independent variables as follows: gender, age, marital status, number of children, education, length of service, service in the company, shift work, working hours, managerial function, and working conditions.

**Table 6:** Factors affecting the PA variable: results of multiple regression analysis.

**Characteristics**		**B**	**S.E**.	**Wald**	**df**	**Sig**.	**Exp (B)**
**Sex**	Female	Reference value					
	Male	0.972	0.562	2.993	1	0.084	2.644
Age		−0.003	0.010	0.121	1	0.728	0.997
Marital status	Married	Reference value					
	Unmarried	0.065	0.266	0.059	1	0.808	1.067
	Extramarital union	0.370	0.602	0.377	1	0.539	1.448
	Divorced	0.444	0.444	1.002	1	0.317	1.559
	Widower/widow	0.706	1.010	0.489	1	0.484	2.027
Number of children	None	Reference value					
	One	−0.124	0.300	0.170	1	0.680	0.884
	Two	−0.124	0.267	0.214	1	0.643	0.884
	Three	−0.050	0.506	0.010	1	0.922	0.952
Education	Three years of high school	Reference value					
	Four years of high school	0.049	0.263	0.035	1	0.852	1.050
	High school—two and a half year studies	1.308	0.559	5.482	1	0.019	3.698
	High school−3-year vocational or academic studies	0.797	0.575	1.919	1	0.166	2.219
	Faculty—basics or master studies	0.422	0.446	0.897	1	0.344	1.525
Length of service		−0.004	0.009	0.137	1	0.711	0.996
Length of service in company		0.017	0.025	0.491	1	0.484	1.018
Work in shifts	No	Reference value					
	Yes	0.124	0.293	0.179	1	0.672	1.132
Work hours	Up to 8 h	Reference value					
	8–12 h	0.234	0.383	0.374	1	0.541	1.264
	More than 12 h	−0.026	0.500	0.003	1	0.958	0.974
Management function	No	Reference value					
	Yes	0.257	0.306	0.704	1	0.401	1.293
Work conditions	Satisfied	Reference value					
	Neither satisfied nor dissatisfied	0.188	0.244	0.593	1	0.441	1.207
	Dissatisfied	0.628	0.481	1.705	1	0.192	1.874

Table 7 shows the results of the dependent “total burnout” modeling with the application of multivariate logistic regression analysis in regard to the independent variables as follows: gender, age, marital status, number of children, education, length of service, service in the company, shift work, working hours, managerial function, and working conditions.

**Table 7:** Factors affecting the Total Burnout: results of multiple regression analysis.

**Characteristics**		**B**	**S.E**.	**Wald**	**df**	**Sig**.	**Exp (B)**
Sex	Female	Reference value					
	Male	−1.195	0.751	2.532	1	0.112	0.303
Age		0.006	0.011	0.254	1	0.614	1.006
Marital status	Married	Reference value					
	Unmarried	0.130	0.293	0.198	1	0.657	1.139
	Extramarital union	0.115	0.685	0.028	1	0.867	1.122
	Divorced	−1.138	0.757	2.261	1	0.133	0.321
	Widower/widow	1.214	1.012	1.437	1	0.231	3.365
Number of children	None	Reference value					
	One	−0.303	0.350	0.750	1	0.386	0.738
	Two	0.035	0.295	0.014	1	0.907	1.035
	Three	−0.243	0.598	0.165	1	0.685	0.784
Education	Three years of high school	Reference value					
	Four years of high school	0.027	0.292	0.008	1	0.927	1.027
	High school—two and a half year studies	0.148	0.624	0.056	1	0.813	1.159
	High school−3-year vocational or academic studies	−0.053	0.693	0.006	1	0.939	0.949
	Faculty—basics or master studies	0.197	0.499	0.156	1	0.693	1.217
Length of service		0.005	0.011	0.244	1	0.621	1.005
Length of service in company		0.021	0.028	0.565	1	0.452	1.021
Work in shifts	No	Reference value					
	Yes	−1.386	0.310	19.98	1	0.000	0.250
Work hours	Up to 8 h	Reference value					
	8–12 h	0.917	0.549	2.794	1	0.095	2.502
	More than 12 h	1.110	0.638	3.030	1	0.082	3.034
Management function	No	Reference value					
	Yes	−0.020	0.356	0.003	1	0.954	0.980
Work conditions	Satisfied	Reference value					
	Neither satisfied nor dissatisfied	0.428	0.275	2.420	1	0.120	1.534
	Dissatisfied	1.337	0.490	7.457	1	0.006	3.809

The dependent variable “total burnout” was transformed into a binary type. The reference category is represented by low and moderate level of total burnout.

A respondent who works in shifts has 75% increased probability to manifest the total burnout in comparison to respondents who do not work in shifts.

Respondents working 8–12 h have 150% greater probability to manifest the total burnout, and respondents working more than 12 h have 203% greater probability to manifest the total burnout.

A respondent who was not satisfied with the working conditions has 280% greater probability to manifest the total burnout in comparison to the respondents who are satisfied with the working conditions.

## Discussion

We have tested the factors that lead to the development of burnout syndrome in employees of privately-owned security agencies in Central Serbia. A multicentric cross-sectional study was applied which included a representative number of employees from seven privately-owned security agencies. Data was collected using specific MBI-HSS questionnaire.

According to the obtained results, younger age, female gender, shorter working experience, shift work, shifts longer than 12 h a day as well as 8–12 h shifts and dissatisfaction with working conditions were significant factors for the development of burnout syndrome in employees of private security agencies.

More than 90% of participants in our study were men. Women were significantly younger than men and had a significantly shorter work experience compared to men. More than 80% of participants worked in shifts and almost the same number worked more than 8–12 h a day.

According to the results of multivariate analysis, the employees who worked in shifts had 75% more risk of burnout syndrome. The highest risk of developing burnout was observed in employees who worked 12 h a day as well as those who worked 8–12 h a day.

Two thirds of participants were satisfied with their working conditions and a little less than one third did not answer this question. The results of multivariate analysis showed that the employees who were not satisfied with the working conditions had a higher risk of developing burnout but the risk was also higher, by 50%, in employees who did not answer this question.

The results obtained by our study are in accordance with results in relevant literature (23, 24). According to the results of a study conducted in Brazil, young employers with lower level of education and shorter length of service had bigger emotional stress at work and higher EE, and the length of service and age were indicated as significant predictors of the total syndrome. Educated individuals with more work experience have more self-confidence in the performance of their duties; they show greater self-control in contact with various stressors and experience less emotional trauma (10).

Results from meta-analysis showed gender differences in burnout across a wide range of occupations found that women reported significantly higher levels of emotional exhaustion (*k* = 199, *d* = 0.09), and men reported significantly higher levels of depersonalization (*k* = 184, *d* = −0.19) (25). Meta-analytic results have also indicated a negative relationship between age and emotional exhaustion (*k* = 34, *r* = −0.16) (23).

In our study, sex and education level were significant factors which impacted the PA scale. Men and those employees who had higher education level had a lower risk of feeling unfulfilled, compared to women and employees who graduated from a 3-year high school. Our results are in agreement with the other literature data (10, 23–25).

Considering the scores of each subscale, we have noticed a significant burnout syndrome level among professional staff who provides security of individuals and their property in private Agencies in Central Serbia.

86.1% of respondents had high and moderate EE levels. The largest number of the respondents had high DP levels−82.4%, 16.1% of respondents had moderate levels while 1.4% of respondents had low DP levels.

According to Maslach theory, EE has had the greatest prevalence in surveys (2) as well as in our study. Similar findings can find in paper of other authors (25–27).

In our study, 34.6% of participants had low levels of PA, 32.9% had moderate levels and 32.6% had high levels of PA. Our results are in accordance with results in relevant literature (26, 27).

According to Mastracci et al. use-of-force is a workplace stressor (27). Less than a third of our participants were obliged to carry a weapon at the work place and this was not a significant factor for the development of burnout syndrome. However, different results are available in literature.

Results of a study performed in the professional military personnel of Serbian Armed Forces (28) showed that the highest level of burnout was measured on the subscales Emotional exhaustion (EE) in military personnel aged 23–30 years of age (*p* < 0.05) and anxiety increased with age (*p* < 0.001). Total scores on the subscales EE and DP increased, while on the subscale PA decreased with the increase of the total score of Back Anxiety Inventory Scale (*p* < 0.001).

## Conclusion

Burnout can be response to chronic job stress for working staff in seven private agencies for security in Central Serbia. According to the results obtained in our study, the most significant factors for the development of burnout syndrome were female gender, younger age, shorter work experience, working in shifts, working 12 h a day, and more than 8–12 h a day as well as dissatisfaction with working conditions. 86.1% of respondents had high and moderate Emotional Exhaustion levels. The largest number of the respondents had high Depersonalisation levels−82.4%, 16.1% of respondents had moderate levels while 1.4% of respondents had low levels. Factors that significantly reduce probability that working staff will manifest the burnout syndrome in the form of lowered Personal Accomplishment level were male sex and “college”-University education. The results of the study indicate that further studies are needed among professional staff who provide security of individuals and their property in private Agencies in Central Serbia.

### The Strength of the Study Are

This was the first study among professional staff who provide security of individuals and their property in private Agencies in Central Serbia and it was conducted on the representative sample of employees in seven sities. The study named the most important predictors for **burnout syndrome and showed that there is a place for preventive**.

### Our Study Has Some Limitations

There were a small number of women in our sample, because in our country, yet, a small number of women work this job. We couldn't include employees from private agencies from the two provinces, because we didn't get official permissions.

## Data Availability Statement

The raw data supporting the conclusions of this article will be made available by the authors, without undue reservation.

## Ethics Statement

The studies involving human participants were reviewed and approved by The Ethics Committee of the Faculty of Medicine of the University of Pristina with temporary seat in Kosovska Mitrovica by the Decision No 09-972-1 dated September 10, 2018. The patients/participants provided their written informed consent to participate in this study. Written informed consent was obtained from the individual(s) for the publication of any potentially identifiable images or data included in this article.

## Author Contributions

DV wrote the manuscript, organized data base, and did investigation. NR performed statistical analysis. MRM, NR, and LK contribute to conception and design of the study. DV, VS, DI, and MS did the investigation. LS, MVM, and SMÐ wrote sections of the manuscript. All authors contributed to manuscript revision, read, and approved the submitted version.

## Conflict of Interest

The authors declare that the research was conducted in the absence of any commercial or financial relationships that could be construed as a potential conflict of interest.
